# Concentration Addition, Independent Action and Generalized Concentration Addition Models for Mixture Effect Prediction of Sex Hormone Synthesis *In Vitro*

**DOI:** 10.1371/journal.pone.0070490

**Published:** 2013-08-22

**Authors:** Niels Hadrup, Camilla Taxvig, Mikael Pedersen, Christine Nellemann, Ulla Hass, Anne Marie Vinggaard

**Affiliations:** 1 Division of Toxicology and Risk Assessment, National Food Institute, Technical University of Denmark, Søborg, Denmark; 2 Division of Food Chemistry, National Food Institute, Technical University of Denmark, Søborg, Denmark; Indian Institute of Toxicology Reserach, India

## Abstract

Humans are concomitantly exposed to numerous chemicals. An infinite number of combinations and doses thereof can be imagined. For toxicological risk assessment the mathematical prediction of mixture effects, using knowledge on single chemicals, is therefore desirable. We investigated pros and cons of the concentration addition (CA), independent action (IA) and generalized concentration addition (GCA) models. First we measured effects of single chemicals and mixtures thereof on steroid synthesis in H295R cells. Then single chemical data were applied to the models; predictions of mixture effects were calculated and compared to the experimental mixture data. Mixture 1 contained environmental chemicals adjusted in ratio according to human exposure levels. Mixture 2 was a potency adjusted mixture containing five pesticides. Prediction of testosterone effects coincided with the experimental Mixture 1 data. In contrast, antagonism was observed for effects of Mixture 2 on this hormone. The mixtures contained chemicals exerting only limited maximal effects. This hampered prediction by the CA and IA models, whereas the GCA model could be used to predict a full dose response curve. Regarding effects on progesterone and estradiol, some chemicals were having stimulatory effects whereas others had inhibitory effects. The three models were not applicable in this situation and no predictions could be performed. Finally, the expected contributions of single chemicals to the mixture effects were calculated. Prochloraz was the predominant but not sole driver of the mixtures, suggesting that one chemical alone was not responsible for the mixture effects. In conclusion, the GCA model seemed to be superior to the CA and IA models for the prediction of testosterone effects. A situation with chemicals exerting opposing effects, for which the models could not be applied, was identified. In addition, the data indicate that in non-potency adjusted mixtures the effects cannot always be accounted for by single chemicals.

## Introduction

Most humans are concomitantly exposed to multiple chemicals at any given point in time [1], [2]. Approximately 84,000 chemicals are registered in the Chemical Substance Inventory [3]; hence the potential for combined effects of multiple chemicals is overwhelming. It is impossible to test every chemical combination, therefore it is desirable to be able to predict effects of mixtures from the knowledge on effects of single chemicals. For this purpose, a range of mathematical models have been developed. Concentration addition (CA), also called dose addition, was introduced by Loewe and Muischneck [4]. This model is based on a dilution principle, and was designed for chemicals with a similar mechanism of action, and has proven effective in several settings [5], [6]. Independent action (IA) was first applied to biological data by Bliss [7]. IA is designed for mixtures of chemicals that have distinct mechanisms of action, and its usefulness has been confirmed in several settings [8], [9]. From a practical point of view, it is desirable to be able to use a single model for all situations, also because mechanisms of action are often unknown. Head to head comparisons of CA and IA have been conducted. Even when the models are challenged with chemicals having different mechanisms of action and chemicals mixed according to their potency to exert equal effects, the difference in prediction by IA and CA does not exceed a factor of five [8], [9]. This relatively minor difference suggests that either model may be sufficient for risk assessment purposes. However, both models have a shortcoming in dealing with mixtures having constituents with high potency but low maximal effect (low efficacy). This is because they can only predict up to the maximal effect level of the chemical with the lowest efficacy. To address this, Howard and Webster developed the generalized concentration addition (GCA) model, which is a modification of the CA model [10]. This model has proven effective in calculating mixture effects of aryl hydrocarbon receptor agonists [10], [11].

The H295R cell steroidogenesis assay is suitable for the investigation of prediction models, because multiple chemicals can be tested in a system that has several different enzymes to be concomitantly targeted by chemicals [12]. Thus this cell system can form the basis for investigation of chemicals with distinct mechanisms of action in perturbing steroidogenesis. In the present investigation we utilized the H295R steroidogenesis assay to test pros and cons of the CA, IA and GCA models in predicting effects of chemical mixtures on steroid hormone synthesis. Two mixtures were applied. First, a “real world like mixture” of 12 chemicals designed to reflect a mixture of endocrine active environmental chemicals to which the European population is typically exposed. These are chemicals such as pesticides, phthalate plasticizers, sun filters, the plastic additive bisphenol A, and paraben preservatives; For which information on *in vivo* endocrine disrupting effects was available (Table 1). The ratios of the chemicals in the mixture are determined by the levels of exposure to humans [13]. Second, we applied a “potency adjusted mixture” encompassing five pesticides, with ratios adjusted in order for the single components to have equal effects on mammals in terms of no observed adverse effect levels (NOAELs) on the endpoint gestation length [14] (Table 1). The steroid synthesis capacity of the human adrenocortical carcinoma cell line, H295R, was investigated for Mixture 1. Out of eight measured hormones, progesterone, testosterone and estradiol were selected for in depth investigations of mixtures and single chemicals. This selection was based partly on their importance in human physiology and partly on their ability to be regulated by the mixture. Dose-response data on these three hormones obtained with single chemicals were next applied to the mixture models. Finally the obtained mixture predictions were compared to the experimental data of the mixtures.

**Table 1:** Details of test chemicals.

CAS registry number	Chemical name	Use	Ratio in mixture (weight)
***Mixture 1***			
80-05-7	bisphenol A	plastic additive	0.005
94-26-8	butyl paraben	preservative	0.26
84-74-2	dibutylphtalate (DBP)	plasticizer	0.030
117-81-7	bis(2-ethylhexyl)phthalate (DEHP)	plasticizer	0.043
36861-47-9	4-methylbenzylidene camphor (4-MBC)	sun filter	0.19
5466-77-3	2-Ethylhexyl-4-methoxycinnamate (OMC)	sun filter	0.34
72-55-9	dichlorodiphenyldichloroethylene (DDE)	pesticide	0.003
133855-98-8	epoxiconazole	pesticide	0.025
330-55-2	linuron	pesticide	0.002
67747-09-5	prochloraz	pesticide	0.031
32809-16-8	procymidone	pesticide	0.044
50471-44-8	vinclozolin	pesticide	0.026
***Mixture 2***			
133855-98-8	epoxiconazole	pesticide	0.09
8018-01-7	mancozeb	pesticide	0.06
67747-09-5	prochloraz	pesticide	0.18
32809-16-8	procymidone	pesticide	0.35
107534-96-3	tebuconazole	pesticide	0.32

## Materials and Methods

### Ethics statement

The human H295R cell line used in this study was obtained commercially. Its origin was previously described in a publication from another group [12].

### Cell culture and chemicals

NCI-H295R human adrenocortical carcinoma cells (ATCC no. CRL-2128, LGC Standards, Boras, Sweden) were cultured in DMEM/F12 medium (w/o phenol red) with HEPES (cat. no. 11039021 Life Technologies, Nærum, Denmark) containing 2.5% Nu-Serum (cat. no. 355100, BD Biosciences, Franklin Lakes, NJ, USA) and 1% ITS-aqueous solution containing human recombinant insulin, human transferrin (0.6 mg/mL each), selenous acid (0.6 µg/mL), BSA (0.1 g/mL) and linoleic acid (0.5 mg/mL) (cat. no. 734-1315, BD Biosciences, Franklin Lakes, NJ, USA) in a humidified cell incubator at 37°C with 5% CO_2_. H295R cells were seeded in 24-well plates (cat. no. 734-1212, Corning, Amsterdam, Netherlands) in a volume of 1 mL containing 3×10^5^ cells/well, and left to grow for 24 h. The compositions of the chemical mixtures are described in table 1 (ratios are based on weight). The mixture measurements were done with a fixed ratio design in which the ratio of individual chemicals in the mixtures were kept constant, whereas the overall concentration of the mixtures were varied. Chemicals were added and left to incubate for 48 h. At the end of the incubation period the supernatant was removed and frozen at −80°C for hormone analyses. Single chemicals and the mixtures were tested at concentrations ranging from 0.04 to 30 µM (n = 3 per concentration). For estradiol, progesterone and testosterone additional independent experiments in triplicates were conducted to assess whether obtained effects were consistent.. For an evaluation of cytotoxicity, cells were added 5 mg/mL MTT (3-(4,5-Dimethylthiazol-2-yl)-2,5-diphenyltetrazolium bromide) (cat. no. M2128, Sigma, St. Louis, USA) and incubated for approximately 1.5 h at 37°C at 5% CO_2_. Medium was next removed, 0.5 mL isopropanol was added and contents were mixed for 5 min on a plate shaker. Fluorescence was next measured on a plate reader (Wallac Victor2 1420 Multilabel Counter, Perkin Elmer, Massachusetts, USA) at a wavelength of 570 nm with a 630 nm reference to negate effects of cell debris.

### Hormone measurements

The following hormones were measured by LC-MS/MS: Progesterone, dehydroepiandrosterone, estrone and testosterone (standards were obtained from: Sigma-Aldrich, Copenhagen, Denmark) and 17-OH-progesterone (Steraloids, Rhode Island, USA), cortisol (Riedel-de Häen, Seelze, Germany) and androstenedione (Cerilliant, Round Rock, USA). Hormones were measured as previously described [15]. Briefly, supernatant was extracted with a C18 end-capped SPE cartridge (500 mg, 3 ml) (Merck, Darmstadt, Germany) after the addition of an internal standard solution of testosterone-d2, 17β-estradiol-d3 and methyltestosterone-d3. Impurities were next removed from the cartridge with demineralized water followed by elution of steroid hormones from the cartridge with methanol. The extract was next evaporated to dryness using nitrogen, and resuspended in a 40% solution of methanol in demineralised water.

The steroid hormones were separated, detected, and quantified using the LC-MS/MS method as previously described [15]. Minor modifications were made to accommodate more hormones. The LC system (Agilent 1100) was equipped with an Atlantis C18 column (2.1×150 mm, 3 µm) (Waters Corp., Milford, MA, USA) maintained at 40°C. The sample injection volume was 50 µL. Estrone was measured in ESI− mode using 65% methanol and 0.01% ammonia for the mobile phase (0.15 mL/min, isocratic flow rate). The remaining steroids were measured in ESI+ mode using 65% methanol and 0.1% acetic acid for the mobile phase (0.2 mL/min, isocratic flow rate). The mass spectrometer was a Quattro Ultima Triple Quadropole Instrument (Waters Corp., Milford, MA, USA). Calibration standards were run before and after sample analyses at levels of: 0.25, 1.25, 2.5, 5.0 and 10 ng/mL. Chromatograms of the standards are included in the supplementary material (Figure S1). The absolute recoveries of the hormones in cell extracts were estimated to be 70–87%, based on the absolute recoveries of the three internal standards in 90 experiments [15]. The limit of quantification (LOQ) of the cell extracts were estimated as the concentration corresponding to six times signal-to-noise, and was <0.1 ng/mL for all hormones except for dehydroepiandrosterone (LOQ<0.8 ng/mL). Testosterone was quantified as the sum of α and β-testosterone.

Progesterone, testosterone and estradiol were also measured by Dissociation-Enhanced Lanthanide Fluorescent Immunoassay (DELFIA). IST Isolute SPE columns C18, 200 mg, 3 ml (cat.no. 220-0020-B, Mikrolab Aarhus Denmark) were washed with 2.5 mL methanol using vacuum suction, and the columns were washed with 2.5 mL water, samples were diluted with water 1∶1 v/v (800 µL+800 µL) and applied to the column at a maximal flow rate of 1 mL/min, non-steroidal molecules were eluted by washing with 2.5 mL 20% methanol, and steroids were eluted with 2×2.5 ml 100% methanol. The eluate was evaporated for approximately 4½ hr in a centrifugal vacuum concentrator (SpeedVac, Thermo Fisher Scientific, Waltham, MA, USA). Samples were re-suspended in 200 mL Diluent 1 (cat. no. G127-100, Perkin Elmer, Waltham, Massachusetts, USA) and stored at 4°C. Samples were next placed in a water bath for 10 min at 45°C to dissolve the steroid hormones. Estradiol, progesterone and testosterone were then analysed according to the description of the manufacturer (Perkin Elmer, Waltham, Massachusetts, USA, Estradiol: Cat. no. 1244-056, Progesterone: Cat. no. A066.101, Testosterone: Cat. no. 100580592).

### Mathematical modelling and statistics

Data for single substances and mixtures were plotted in an x,y graph with x values being Log10 in Graph Pad Prism (Graph Pad Software, La Jolla, USA). To be eligible for the modelling equations, data were tested for significance. As the number of samples at each measurement point was too low (n = 3) to test for normality using the D'Agostino & Pearson omnibus normality test, the non-parametric Kruskal-Wallis test was used with *p*<0.05 considered significant. Only in cases where chemicals had a significant effect, a dose-response curve fit was established and the EC_50_ value included in the mathematical prediction models. The non-linear regression curve fit applied was a sigmoidal (three-parameter logistic) dose-response fit with the Hill slope set at 1 and the bottom value set at 100% (control level). For stimulatory responses (above 100%), the top value was set to be maximally at the level of the data point with the highest effect. For inhibitory effects (below 100%) the top value was set to be at the data point with the strongest effect (lowest value). The top value and EC_50_, values were transferred to an Excel spread sheet (Microsoft, Seattle, USA) for prediction calculations.

In graphs of the experimental mixture data, all single chemicals having significant effects were illustrated by a calculated contribution. This was done by dividing concentration values, corresponding to specific effect values, with the reciprocal of the ratio of the chemical in the mixture. This shifts the regression line of each chemical to the right along the x-axis to depict the contribution of the chemical to the mixture effect at each mixture concentration point.

CA was modelled by formula 1.
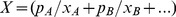
(1)Where X is the concentration of the mixture at which a specific effect occurs. p_A_ is the fraction of chemical A in the mixture and so-forth for chemical B etc.; x_A_ is the concentration level at which chemical A on its own exerts this specific effect. For a range of effect levels x values were calculated, and a prediction curve was established.

IA was modelled by formula 2.
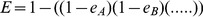
(2)E is the effect of the mixture at a specific concentration; e_A_ is the effect of chemical A at that specific concentration and so-forth for chemical B etc.. For a range of concentration points effects (E) were calculated, and a prediction curve was established.

GCA was modelled by formula 3.
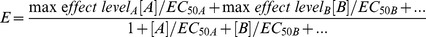
(3)E is the effect of the mixture at a specific concentration. ‘max effect level A’ is the maximal effect level of chemical A, [A] is the concentration of A in the mixture at a specific mixture concentration, EC_50A_ is the EC_50_ value of A and so-forth for chemical B etc.. Thus for a range of mixture concentrations, effect values (E) were calculated using this equation, and a curve was established.

The mathematical models were applied only in situations where effective chemicals in a mixture either all exerted stimulatory effects or all exerted inhibitory effects; Thus if a mixture consisted of chemicals having a stimulatory effect and concomitantly other chemicals having an inhibitory effect, then no predictions were calculated.

## Results

### Effects of Mixture 1 on eight steroid hormones

**Figure 1:**
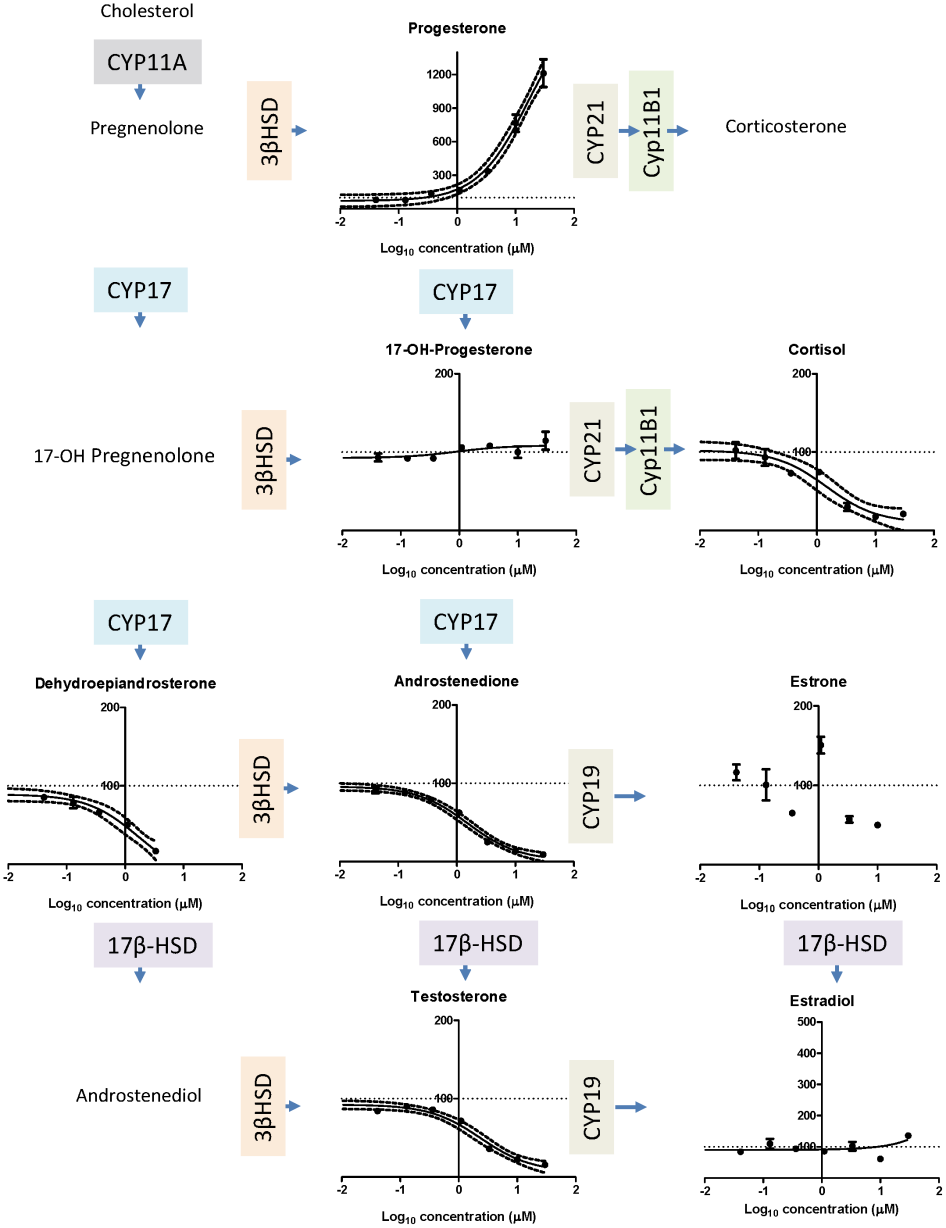
The effect of Mixture 1 on steroid hormone levels in H295R cells. Cells were incubated with Mixture 1 at concentrations ranging from 0.04 to 30 µM for 48 h. Hormones levels were measured by LC-MS/MS, except for estradiol that was measured by DELFIA. The figure shows the results of progesterone, 17-OH-progesterone, cortisol, dehydroepiandrosterone, androstenedione, estrone, testosterone and estradiol arranged according to appropriate steps in steroidogenesis. Data are mean ± SD expressed as per-cent of the control level. A *p*-value of less than 0.05 was considered significant, and in case of significance a sigmoidal curve fit (black line) was applied with a 95% confidence band (black dotted lines). Enzymes involved in steroidogenesis are illustrated by colour shaded boxes at appropriate steps. Abbreviations are as follows: CYP: Cytochrome P450, HSD: Hydroxysteroid dehydrogenase.

By LC-MS/MS the following was found for Mixture 1 (Fig. 1): Progesterone levels were increased with an EC_50_ value of 16 µM and a measured maximal effect level of 1200% of control. It should be noted that the curve did not seem to have reached its maximal effect level, thus the value is a tentative E_max_ value, 17α-OH progesterone was unchanged. Cortisol was decreased with an EC_50_ value of 1.5 µM and a measured maximal effect level of 18% of control; dehydroepiandrosterone was decreased with an EC_50_ value of 0.43 µM and a measured maximal effect level at 17% of control. Androstenedione was decreased with an EC_50_ value of 1.5 µM and a measured maximal effect level of 9% of control. Testosterone was also decreased with an EC_50_ value of 2.4 µM and a measured maximal effect level of 16% of control (Fig. 1). Estrone was unchanged. Estradiol was measured by DELFIA as it was not detected by LC-MS/MS. Mixture 1 had no effect on the estradiol level (Fig. 1).

### Effects of Mixture 1 and its constituents on progesterone, testosterone and estradiol

**Figure 2:**
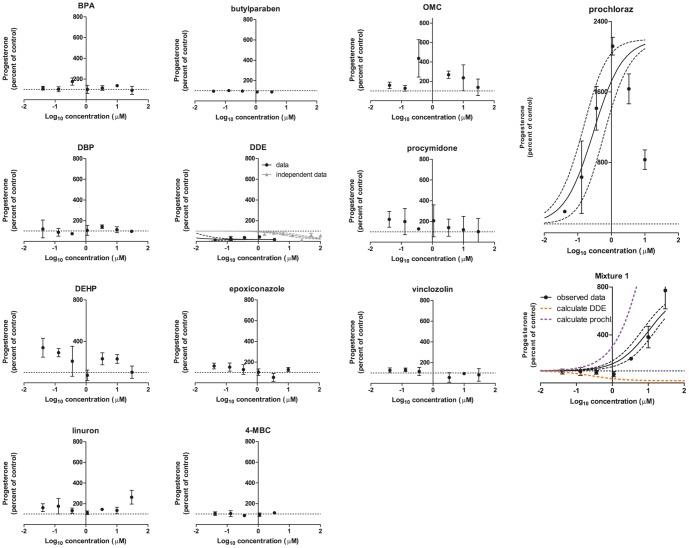
The effect of Mixture 1 and its constituents on progesterone levels in H295R cells. H295R cells were incubated with chemicals or Mixture 1 at concentrations ranging from 0.04 to 30 µM for 48 h. The cell medium was next isolated and progesterone was measured by DELFIA. Data are mean ± SD. A *p*-value of less than 0.05 was considered significant, and in case of significance a sigmoidal curve fit (black line) was applied with a 95% confidence band (black dotted lines). The calculated contribution of each chemical is illustrated on the graph of the mixture data (abbreviated as “calculate” in the graph). This contribution is established by shifting the regression line of single chemical effects to the right along the x-axis by the reciprocal of its ratio in the mixture.

The effects of Mixture 1 and its constituent chemicals on progesterone measured using DELFIA were (Fig. 2): A decrease in progesterone was seen for DDE in the modelled data set (EC_50_: 0.002 µM, maximal effect level (E_max_): 18% of control) and in an independent dataset (EC_50_: 14 µM, E_max_: 16%). An increase in progesterone was found for prochloraz (EC_50_: 0.30 µM, E_max_: 2200%), as well as for Mixture 1 (EC_50_: 10 µM, E_max_: 770%). No effect on progesterone levels was found for BPA, butylparaben, DBP, DEHP, epoxiconazole, linuron, 4-MBC, OMC, procymidone or vinclozolin (full names of abbreviated chemicals are found in table 1).

**Figure 3:**
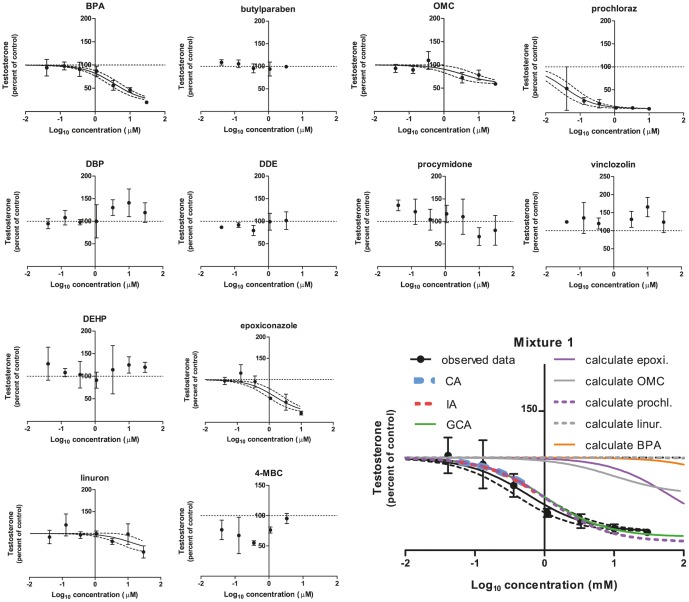
The effect of Mixture 1 and its constituents on testosterone levels in H295R cells. H295R cells were incubated with chemicals or Mixture 1 at concentrations ranging from 0.04 to 30 µM for 48 h. The cell medium was next isolated and testosterone was measured by DELFIA. Data are mean ± SD. A *p*-value of less than 0.05 was considered significant, and in case of significance a sigmoidal curve fit (black line) was applied with a 95% confidence band (black dotted lines). Concentration addition (CA, dotted blue line), independent action (IA, dotted red line) and generalized concentration addition (GCA, green line) predictions were calculated and applied to the graph of the mixture data. The calculated contribution of each chemical is illustrated on the graph of the mixture data (abbreviated as “calculate” in the graph). This contribution is established by shifting the regression line of single chemical effects to the right along the x-axis by the reciprocal of its ratio in the mixture.

Effects on testosterone of the single chemicals and Mixture 1 were (Fig. 3): A decrease in testosterone was seen with BPA (EC_50_: 3.5 µM, E_max_: 20%), epoxiconazole (EC_50_: 1.5 µM, E_max_: 21%), linuron (EC_50_: 13 µM, E_max_: 58%), OMC (EC_50_: 3.4 µM, E_max_: 60% of control), prochloraz (EC_50_: 0.04 µM, E_max_: 9%), and the mixture (EC_50_: 0.6 µM, E_max_: 20%). Butylparaben, DBP, DDE, DEHP, 4-MBC, procymidone, and vinclozolin had no effect.

**Figure 4:**
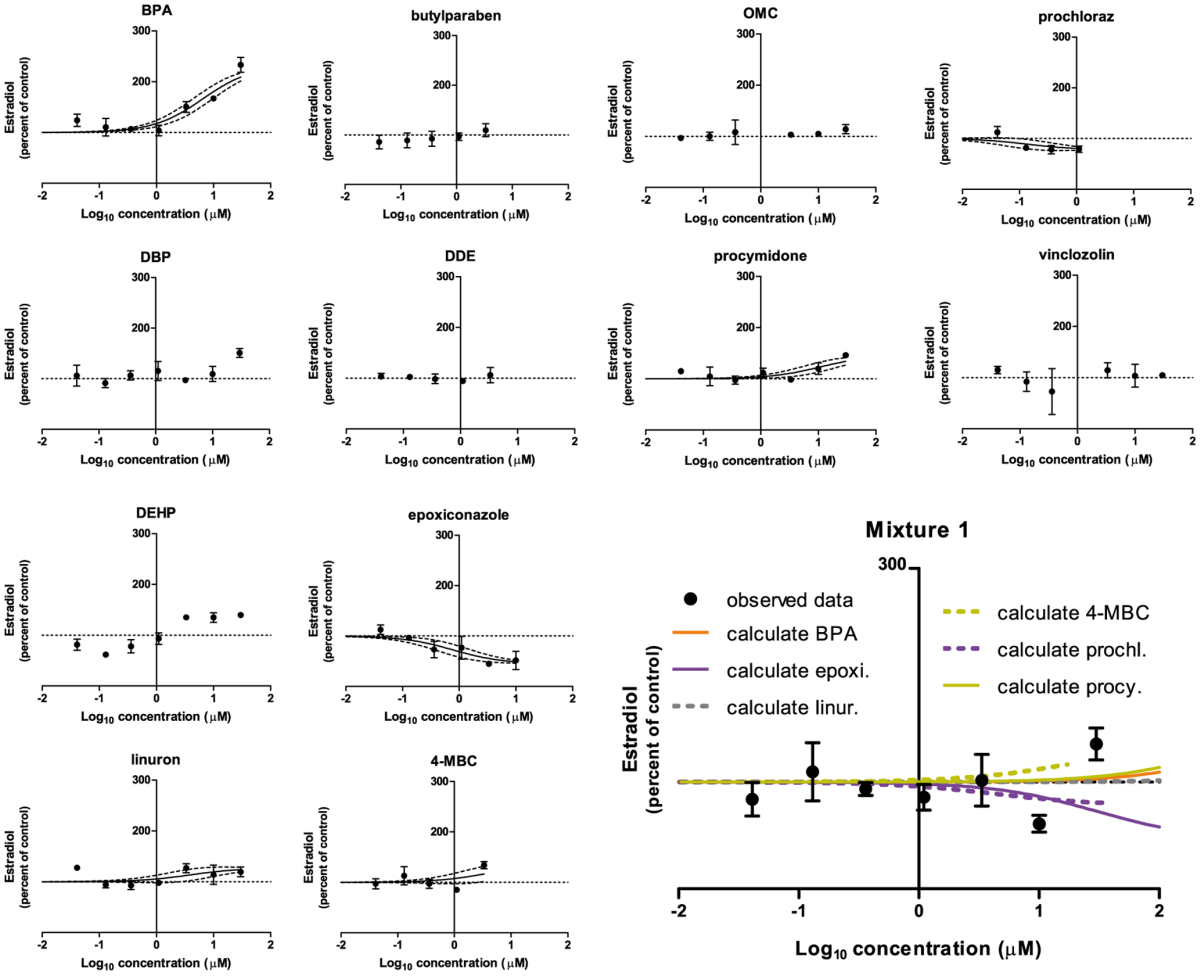
The effect of Mixture 1 and its constituents on estradiol levels in H295R cells. H295R cells were incubated with chemicals or Mixture 1 in concentrations ranging from 0.04 to 30 µM for 48 h. The cell medium was next isolated and estradiol was measured by DELFIA. Data are mean ± SD. A *p*-value of less than 0.05 was considered significant, and in case of significance a sigmoidal curve fit (black line) was applied with a 95% confidence band (black dotted lines). The calculated contribution of each chemical is illustrated on the graph of the mixture data (abbreviated as “calculate” in the graph). This contribution is established by shifting the regression line of single chemical effects to the right along the x-axis by the reciprocal of its ratio in the mixture.

The effects on estradiol were (Fig. 4): An increase as seen with BPA (EC_50_: 6.6 µM, E_max_: 230%), linuron (EC_50_: 4.0 µM, E_max_: 127%), and procymidone (EC_50_: 11 µM, E_max_: 146%). In the presented dataset 4-MBC also showed an increase in estradiol (EC_50_: 3.5 µM, E_max_: 134%); However, this effect was not reproducible and was considered a chance finding. A decrease in the estradiol level was found for epoxiconazole (EC_50_: 0.8 µM, E_max_: 45%), and prochloraz (EC_50_: 0.13 µM, E_max_: 78%). For butylparaben, DBP, DDE, OMC, vinclozolin, and Mixture 1, no effects were found. DEHP showed an effect, but in the included dataset with a non-monotonous dose-response curve. This effect was not seen in an independent experiment.

### Effects of Mixture 2 and its constituents on progesterone, testosterone and estradiol

**Figure 5:**
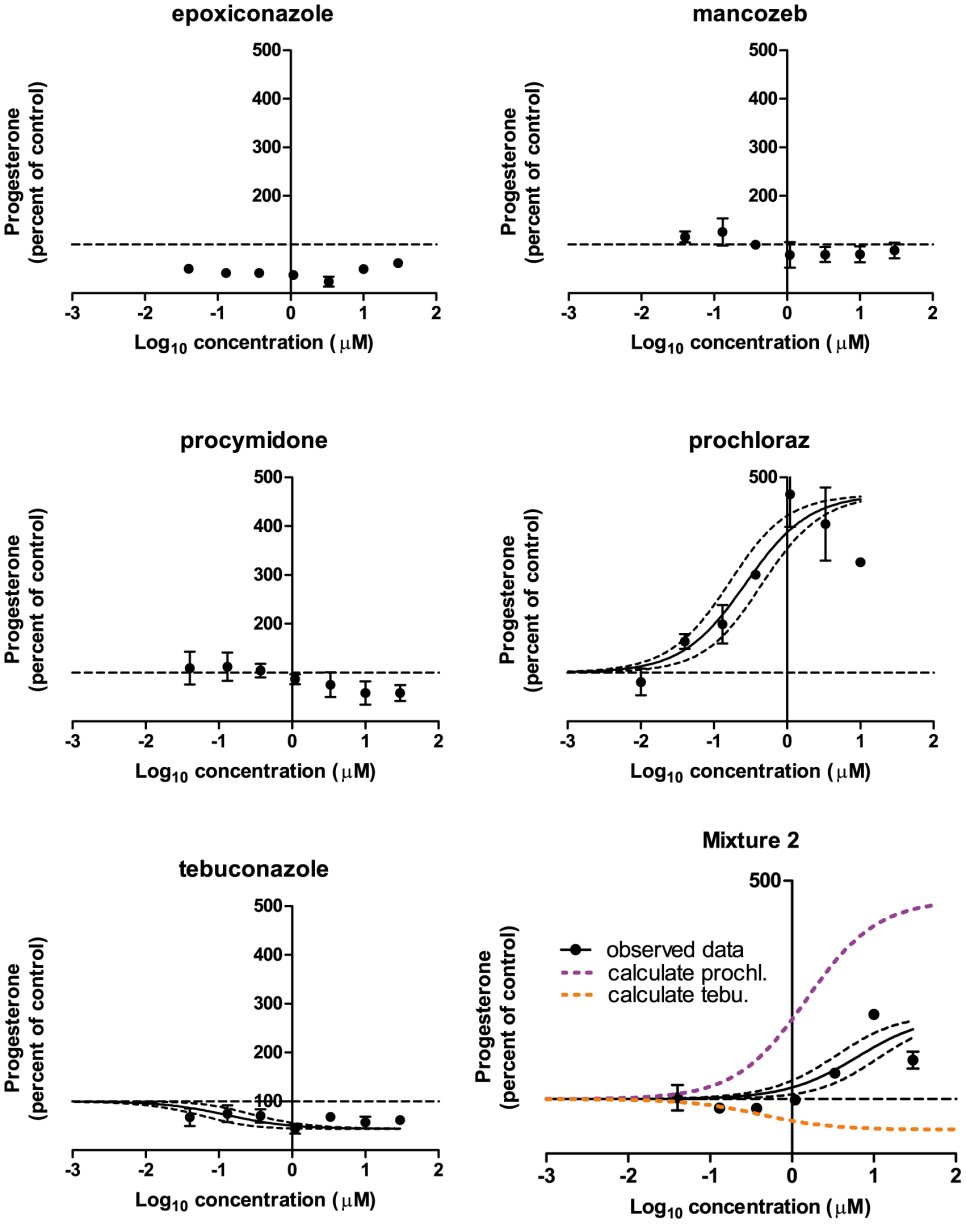
The effect of Mixture 2 and its constituents on progesterone levels in H295R cells. H295R cells were incubated with chemicals or Mixture 2 in concentrations ranging from 0.04 to 30 µM for 48 h. The cell medium was next isolated and progesterone was measured by DELFIA. Data are mean ± SD. A *p*-value of less than 0.05 was considered significant, and in case of significance a sigmoidal curve fit (black line) was applied with a 95% confidence band (black dotted lines). The calculated contribution of each chemical is illustrated on the graph of the mixture data (abbreviated as “calculate” in the graph). This contribution is established by shifting the regression line of single chemical effects to the right along the x-axis by the reciprocal of its ratio in the mixture.

The effects of Mixture 2 and its constituent chemicals on progesterone levels in the H295R cells were as follows (Fig. 5): Tebuconazole decreased progesterone (EC_50_: 0.13 µM, E_max_: 44%). Increased progesterone was found for prochloraz (EC_50_: 0.27 µM, E_max_: 465%) and Mixture 2 (EC_50_: 6.3 µM, E_max_: 255%). Mancozeb, procymidone, and, when taking into consideration an abnormally high control level, epoxiconazole had no effect.

**Figure 6:**
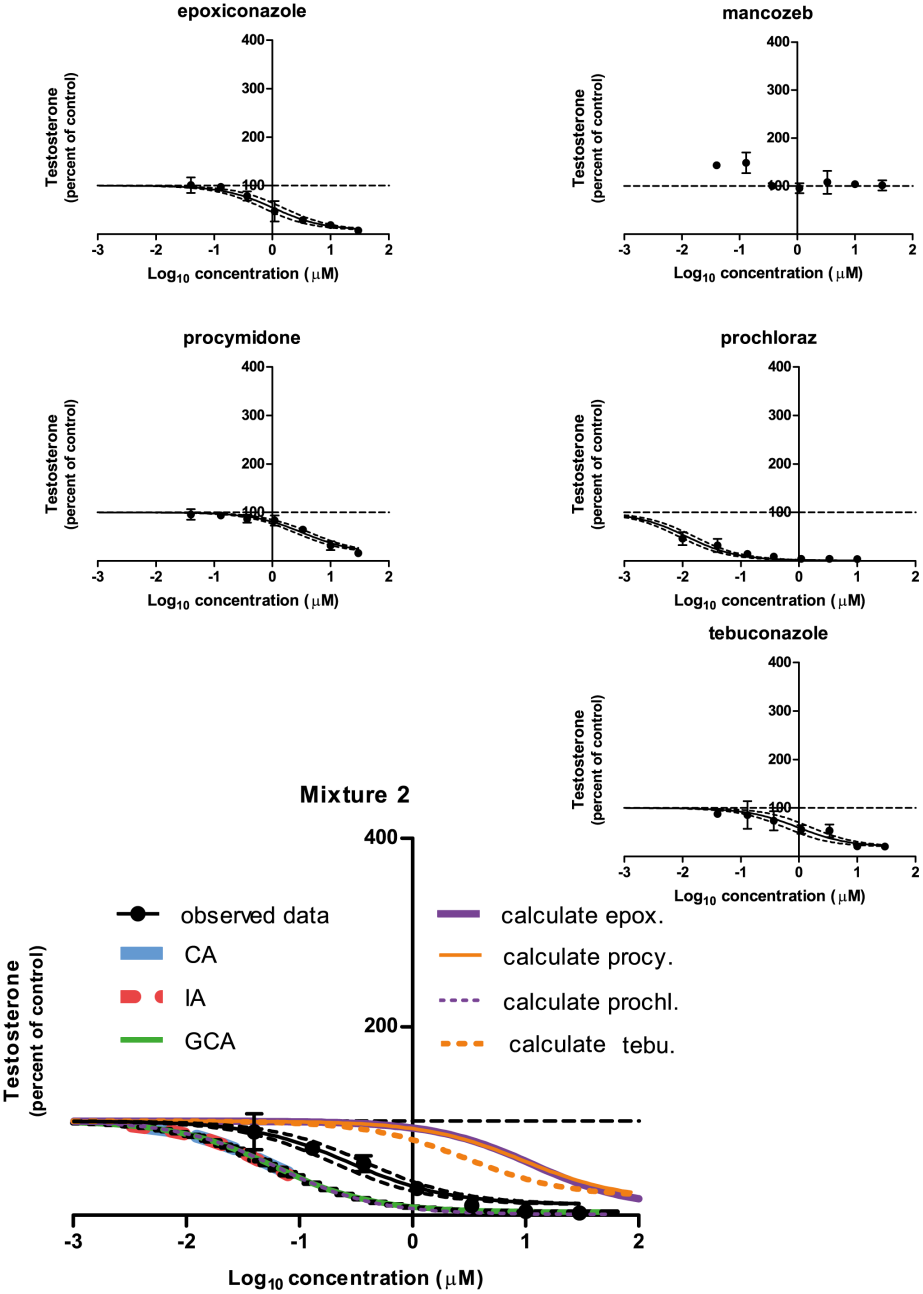
The effect of Mixture 2 and its constituents on testosterone levels in H295R cells. H295R cells were incubated with chemicals or Mixture 2 in concentrations ranging from 0.04 to 30 µM for 48 h. The cell medium was next isolated and estradiol was measured by DELFIA. Data are mean ± SD. A *p*-value of less than 0.05 was considered significant, and in case of significance a sigmoidal curve fit (black line) was applied with a 95% confidence band (black dotted lines). Concentration addition (CA, dotted blue line), independent action (IA, dotted red line) and generalized concentration addition (GCA, green line) predictions were calculated and applied to the graph of the mixture data. The calculated contribution of each chemical is illustrated on the graph of the mixture data (abbreviated as “calculate” in the graph). This contribution is established by shifting the regression line of single chemical effects to the right along the x-axis by the reciprocal of its ratio in the mixture.

For testosterone, effects of Mixture 2 and its constituent chemicals the following was found (Fig. 6): A decrease in testosterone was observed for epoxiconazole (EC_50_: 1.0 µM, E_max_: 8%), procymidone (EC_50_: 3.4 µM, E_max_: 16%), prochloraz (EC_50_: 0.011 µM, E_max_: 1%), and tebuconazole (EC_50_: 0.011 µM, E_max_: 44%). For Mixture 2, a decrease in testosterone was also seen (EC_50_: 0.28 µM, E_max_: 12%). Mancozeb had no effect.

**Figure 7:**
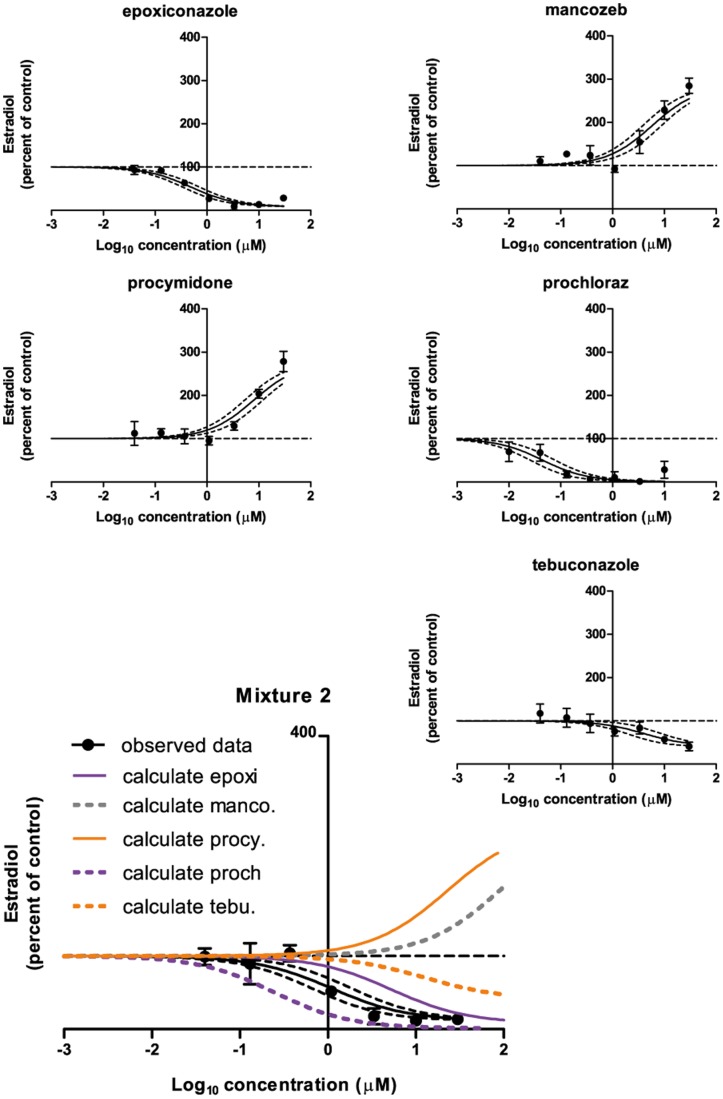
The effect of Mixture 2 and its constituents on estradiol levels in H295R cells. H295R cells were incubated with chemicals or Mixture 2 in concentrations ranging from 0.04 to 30 µM for 48 h. The cell medium was next isolated and estradiol was measured by DELFIA. Data are mean ± SD. A *p*-value of less than 0.05 was considered significant, and in case of significance a sigmoidal curve fit (black line) was applied with a 95% confidence band (black dotted lines). The calculated contribution of each chemical is illustrated on the graph of the mixture data (abbreviated as “calculate” in the graph). This contribution is established by shifting the regression line of single chemical effects to the right along the x-axis by the reciprocal of its ratio in the mixture.

The effects of Mixture 2 and its constituent chemicals on estradiol were the following (Fig. 7): A decrease was found for epoxiconazole (EC_50_: 0.48 µM, E_max_: 8%), prochloraz (EC_50_: 0.044 µM, E_max_: 1%), tebuconazole (EC_50_: 4.0 µM, E_max_: 41%) and Mixture 2 (EC_50_: 1.1 µM, E_max_: 12%). Increased estradiol levels were found for mancozeb (EC_50_: 5.7 µM E_max_: 284%) and procymidone (EC_50_: 8.0 µM, E_max_: 278%).

### Mixture effect predictions obtained with CA, IA and GCA modeling

Regarding Mixture 1, the DA and IA model yielded a prediction line for the testosterone data in the range of 100 to 60% of control that was contained within the 95% confidence belt of the experimental data for the mixture (Fig. 3). The GCA model yielded a prediction line that covered the whole range of the experimental data (100 to 20% of control) and that was contained within the 95% confidence belt of the data (Fig. 3). For progesterone and estradiol no predictions could be established due to the mixtures consisting both of chemicals having stimulatory effects and chemicals having inhibitory effects.

Regarding Mixture 2, The DA and IA model yielded a prediction line for the testosterone data in the range of 100 to 44% of control that was located to the left of the 95% confidence belt of the experimental data for the mixture (Fig. 6). The GCA model yielded a prediction line that covered the whole range of the experimental data (100 to 12% of control) also located to the left of the 95% confidence belt of the experimental data for the mixture (Fig. 6). Again, for progesterone and estradiol, no predictions could be established due to the mixtures containing chemicals with opposing effects. Regarding calculated contributions of each chemical in the mixtures, prochloraz was located to the left of all experimental data dose-response curves except for Mixture 1 testosterone. Here the calculated contribution was contained within the 95% confidence band of the experimental mixture data. For all other chemicals having effects, the calculated contribution was located to the right of the dose-response curves of the experimental mixture data.

## Discussion

### Application of CA, IA and GCA to in vitro sex hormone data

Regarding the effect of Mixture 1 on testosterone, the predictions of all mathematical models were contained within the 95% confidence band of the experimental data (Fig. 2). In addition, the calculated contribution from prochloraz in the mixture coincided with the mixture prediction. This suggests that prochloraz drives the prediction models as well as the effect of the mixture. This is in accordance with the mathematical nature of the models. The CA and IA models are driven by a single chemical if this single chemical exists in a concentration not very different from the other chemicals and has a potency that is substantially higher than the other members of the mixture. In addition, previously published data suggest that it is typically one chemical that drives the effect in a mixture [16], [17]. Prochloraz being highly potent in our investigation is in agreement with previous data from several settings both *in vitro* and *in vivo* [18]–[25].

For the remaining data of this investigation, mixture effects could not be sufficiently predicted by CA, IA or GCA. For testosterone levels following incubation with Mixture 2, the predictions coincided with the calculated contribution of prochloraz, but the predictions were shifted to the left as compared to the experimental data of the mixture. This suggests that an antagonistic effect had occurred. For progesterone and estradiol it was impossible to establish predictions because there were chemicals in the mixture having opposing effects on the hormone levels. Regarding Mixture 2induced effects on progesterone and estradiol, the calculated contribution of prochloraz was located to the left of the experimental data on the dose-response curve (Fig. 5 and 7). This suggests that other chemicals in the mixtures were able to antagonize the effect of prochloraz. These single chemicals: Epoxiconazole, tebuconazole, vinclozolin, linuron, DDE, OMC and BPA exerted effects as expected from previous findings [22], [26]–[32]. Possible mechanisms underlying the ability of these chemicals to interfere with the effect of prochloraz could be: Competition for binding sites on receptor proteins, transporters or enzymes; Upstream effects perturbing the level of substrate for enzymes affected by prochloraz; Downstream effects counteracting the effect of prochloraz. Looking at the effects of Mixture 1 on the steroidogenesis cascade in the H295R cells (Fig. 1), the following data were obtained. Mixture 1 induced an increase in progesterone; decreases in cortisol, androstenedione and testosterone and had no effect on 17-OH progesterone and estradiol. An interpretation of these findings could be that two or more of the following enzymes were affected by constituents in the mixture: cytochrome P450 (CYP)-11B1, -17, -19 or -21. It has been demonstrated that prochloraz can inhibit CYP17, CYP 11A1 and 3β-HSD2 [23], [24], [33]–[35]. This along with tebuconazole and BPA being linked to CYP19 inhibition and for BPA also CYP17A1 inhibition and decreased estradiol metabolism [32], [34]–[36], suggests that more than one of the above mentioned possibilities are in play concomitantly. Apart from lowering androgens, Mixture 1 also reduced the cortisol level (Fig. 1). This effect could be caused by inhibitory effects on CYP21 or CYP11B1 or by increased metabolism of this steroid.

It is noted that Mixture 1 was not designed in accordance to potency of the individual chemicals. Thus its constituents were not adjusted in concentration to exert equal effects. However, we found for progesterone and estradiol that one chemical was not entirely responsible for driving the effect of the mixture. This provides data to suggest that it is not always one chemical that drives the effect of a non-potency adjusted mixture, as could be suggested based on previous findings [16], [17].

### Pros and cons of GCA as compared to CA and IA

In spite of the H295R cell system having multiple enzymatic steps as potential targets for chemicals with dissimilar mechanisms of action, the CA and IA models give rise to similar results in the current study. This reflects that the models give similar results when one chemical (prochloraz) is driving the effect, and is in line with the finding that the difference in prediction of the two models does not exceed a factor of five [8], [9]. For the CA and IA models only part of the testosterone dose-response curve could be predicted (40–60% *vs.* GCA: 80–90% of control). This is due to the fact that there were chemicals in the mixtures having only partial efficacy. In contrast, the GCA model gives rise to a full prediction line because this model is not restricted by the presence of chemicals with limited efficacy. However, the GCA model by definition utilizes Hill slope values set to 1 [10], and therefore has a shortcoming when dealing with dose-response curves that have slopes differing considerably from 1. The Hill slope by definition equals 1, when a monomer binds to one site without cooperativity, whereas when the receptor or ligand has multiple binding/target sites with positive cooperativity then the Hill slope is higher than 1. When there are multiple binding sites with different affinities for the ligand or when there is a negative cooperativity, then the Hill slope is less than 1 [37]. Taking this into account it might very well be that the further downstream from the target of the chemical the measured endpoint is, the greater the risk that the slope of the curve is deviating from 1. This is especially the case when moving away from the simple *in vitro* systems into the more complex *in vivo* models.

For mixture prediction of chemicals, it should be assessed whether the advantage of being able to predict a full prediction line may outweigh the disadvantage of having to use a fixed Hill slope of 1. An alternative option may be to take the CA as well as the GCA prediction into account.

### Shortcomings of current models in dealing with mixtures containing chemicals with opposing effects

In the current investigation four out of six mixture experiments contained chemicals with opposing effects. To our knowledge these data cannot be handled by the present mixture models. Backhaus and co-workers attempted for a mixture data set on natural algae communities, to use data from single chemicals having a non-monotonous dose-response curve and calculate IA using both stimulatory and inhibitory effect values. This gave a prediction line that was more in accordance with the experimental data of the mixture as compared to an approach that excluded stimulatory values from the calculation [8]. However, the authors stated that it was not theoretically correct to conduct such a calculation. The reason is that IA is based on probabilistic reasoning. The effect values correspond to response probabilities, ranging from 0 (no response) to 1 (total response). Therefore, if negative values for single chemical effects are included, IA loses its probabilistic meaning and is then degraded to a simple calculation technique with no broader, theoretical background.

Non-monotonous dose-response curves are also relevant in human toxicology e.g. regarding drugs and alcohol [38]. Mixtures of chemicals having opposing effects, in addition to the present findings, have also been described in drug interactions [39]. Thus there is a lack of models for the prediction of mixture effects in such situations. A question is whether it is safe to develop models in which chemicals with opposing effects are allowed to cancel out the effect of each other when dealing with human risk assessment. If for example one chemical lowers blood pressure and another one increases, it what is then the probability that a combined effect is a cancellation? A solution could be that all effects by chemicals in a mixture should be added meaning that an effect - regardless of it being stimulatory or inhibitory - should be considered as a perturbing effect; thus absolute values could be added. This would yield a conservative risk assessment compared to procedures where chemicals are allowed to cancel out the effect of others.

## Conclusion

In conclusion, prochloraz seems to be the chemical driving the effect on steroidogenesis of two environmental chemical mixtures, although in some cases the presence of other chemicals diminished its expected contribution. Prediction, using the GCA model in the H295R *in vitro* cell system, yielded a curve that could predict a larger range of the dose-response curve as compared to the CA and IA models. All three models predicted combination effects on testosterone levels but had a shortcoming regarding the prediction of mixtures containing both chemicals with stimulatory effects as well as chemicals having inhibitory effects. Only in one out of six endpoints for the two mixtures in the present study, a prediction of an effect within the 95% confidence band was obtained. Mixture 1 was not designed according to potency, yet the mixture effect could not be accounted for by any single chemical regarding effects on estradiol and progesterone, which indicates that one single chemical does not always drive the effect of a non-potency adjusted mixture. Strategies for assessing cumulative effects in heterogeneous data sets need to be discussed and developed.

## Supporting Information

Figure S1 LC-MS/MS Chromatogram of standard samples used to quantify hormone levels. Estrone, progesterone, 17-OH progesterone, cortisol, androstenedione and testosterones were included at concentrations of 1.25 ng/mL. Dehydroepiandrosterone was included at a concentration of 10 ng/mL. Molecular mass of the hormones and their fragments are included in the right hand upper corner of each graph.
(TIF)

## References

[1] Churchill JE, Ashley DL, Kaye WE (2001) Recent chemical exposures and blood volatile organic compound levels in a large population-based sample. ArchEnvironHealth 56: 157–166. 10.1080/00039890109604068 11339680

[2] Woodruff TJ, Zota AR, Schwartz JM (2011) Environmental chemicals in pregnant women in the United States: NHANES 2003–2004. EnvironHealth Perspect 119: 878–885. 10.1289/ehp.1002727 PMC3114826 21233055

[3] United States Environmental Protection A (2012) Toxic Substance Control Act

[4] Loewe S, Muischnek H (1926) Combinated effects I Announcement - Implements to the problem. Naunyn-Schmiedebergs Archiv fur Experimentelle Pathologie und Pharmakologie 114: 313–326.

[5] Hermens J, Canton H, Janssen P, Dejong R (1984) Quantitative Structure Activity Relationships and Toxicity Studies of Mixtures of Chemicals with Anesthetic Potency - Acute Lethal and Sublethal Toxicity to Daphnia-Magna. Aquatic Toxicology 5: 143–154.

[6] Konemann H (1981) Fish toxicity tests with mixtures of more than two chemicals: a proposal for a quantitative approach and experimental results. Toxicology 19: 229–238. 7233447 10.1016/0300-483x(81)90132-3

[7] Bliss CI (1939) The toxicity of poisons applied jointly. Annals of Applied Biology 26: 585–615.

[8] Backhaus T, Arrhenius A, Blanck H (2004) Toxicity of a mixture of dissimilarly acting substances to natural algal communities: predictive power and limitations of independent action and concentration addition. EnvironSciTechnol 38: 6363–6370. 10.1021/es0497678 15597893

[9] Faust M, Altenburger R, Backhaus T, Blanck H, Boedeker W, et al (2003) Joint algal toxicity of 16 dissimilarly acting chemicals is predictable by the concept of independent action. AquatToxicol 63: 43–63. 10.1016/s0166-445x(02)00133-9 12615420

[10] Howard GJ, Webster TF (2009) Generalized concentration addition: a method for examining mixtures containing partial agonists. JTheorBiol 259: 469–477. 10.1016/j.jtbi.2009.03.030 PMC2737481 19345693

[11] Howard GJ, Schlezinger JJ, Hahn ME, Webster TF (2010) Generalized concentration addition predicts joint effects of aryl hydrocarbon receptor agonists with partial agonists and competitive antagonists. EnvironHealth Perspect 118: 666–672. 10.1289/ehp.0901312 PMC2866683 20435555

[12] Bird IM, Hanley NA, Word RA, Mathis JM, McCarthy JL, et al (1993) Human NCI-H295 adrenocortical carcinoma cells: a model for angiotensin-II-responsive aldosterone secretion. Endocrinology 133: 1555–1561. 8404594 10.1210/endo.133.4.8404594

[13] Christiansen S, Kortenkamp A, Axelstad M, Boberg J, Scholze M, et al (2012) Mixtures of endocrine disrupting contaminants modelled on human high end exposures: an exploratory study in rats. IntJAndrol 35: 303–316. 10.1111/j.1365-2605.2011.01242.x 22372636

[14] Hass U, Boberg J, Christiansen S, Jacobsen PR, Vinggaard AM, et al (2012) Adverse effects on sexual development in rat offspring after low dose exposure to a mixture of endocrine disrupting pesticides. ReprodToxicol 34: 261–274. 10.1016/j.reprotox.2012.05.090 22659286

[15] Mortensen SK, Pedersen M (2007) Confirmatory analysis of acetylgestagens in plasma using liquid chromatography-tandem mass spectrometry. AnalChimActa 586: 217–222. 10.1016/j.aca.2006.10.016 17386714

[16] Heindel JJ, Chapin RE, George J, Gulati DK, Fail PA, et al (1995) Assessment of the reproductive toxicity of a complex mixture of 25 groundwater contaminants in mice and rats. FundamApplToxicol 25: 9–19. 10.1006/faat.1995.1035 7601330

[17] Olmstead AW, LeBlanc GA (2005) Toxicity assessment of environmentally relevant pollutant mixtures using a heuristic model. IntegrEnvironAssessManag 1: 114–122. 10.1897/ieam_2004-005r.1 16639893

[18] Blystone CR, Furr J, Lambright CS, Howdeshell KL, Ryan BC, et al (2007) Prochloraz inhibits testosterone production at dosages below those that affect androgen-dependent organ weights or the onset of puberty in the male Sprague Dawley rat. ToxicolSci 97: 65–74. 10.1093/toxsci/kfm004 17234647

[19] Blystone CR, Lambright CS, Howdeshell KL, Furr J, Sternberg RM, et al (2007) Sensitivity of fetal rat testicular steroidogenesis to maternal prochloraz exposure and the underlying mechanism of inhibition. ToxicolSci 97: 512–519. 10.1093/toxsci/kfm055 17369198

[20] Hecker M, Hollert H, Cooper R, Vinggaard AM, Akahori Y, et al (2007) The OECD validation program of the H295R steroidogenesis assay for the identification of in vitro inhibitors and inducers of testosterone and estradiol production. Phase 2: Inter-laboratory pre-validation studies. Environmental Science and Pollution Research 14: 23–30. 21959537 10.1065/espr2007.03.402

[21] Higley EB, Newsted JL, Zhang X, Giesy JP, Hecker M (2010) Assessment of chemical effects on aromatase activity using the H295R cell line. EnvironSciPollutResInt 17: 1137–1148. 10.1007/s11356-009-0285-3 20087668

[22] Kjaerstad MB, Taxvig C, Andersen HR, Nellemann C (2010) Mixture effects of endocrine disrupting compounds in vitro. IntJAndrol 33: 425–433. 10.1111/j.1365-2605.2009.01034.x 20132345

[23] Laier P, Metzdorff SB, Borch J, Hagen ML, Hass U, et al (2006) Mechanisms of action underlying the antiandrogenic effects of the fungicide prochloraz. ToxicolApplPharmacol 213: 160–171. 10.1016/j.taap.2005.10.013 16375936

[24] Rijk JC, Peijnenburg AA, Blokland MH, Lommen A, Hoogenboom RL, et al (2012) Screening for modulatory effects on steroidogenesis using the human H295R adrenocortical cell line: a metabolomics approach. ChemResToxicol 25: 1720–1731. 10.1021/tx3001779 22768806

[25] Vinggaard AM, Christiansen S, Laier P, Poulsen ME, Breinholt V, et al (2005) Perinatal exposure to the fungicide prochloraz feminizes the male rat offspring. ToxicolSci 85: 886–897. 10.1093/toxsci/kfi150 15788727

[26] Chedrese PJ, Feyles F (2001) The diverse mechanism of action of dichlorodiphenyldichloroethylene (DDE) and methoxychlor in ovarian cells in vitro. ReprodToxicol 15: 693–698. 10.1016/s0890-6238(01)00172-1 11738522

[27] Hotchkiss AK, Parks-Saldutti LG, Ostby JS, Lambright C, Furr J, et al (2004) A mixture of the “antiandrogens” linuron and butyl benzyl phthalate alters sexual differentiation of the male rat in a cumulative fashion. BiolReprod 71: 1852–1861. 10.1095/biolreprod.104.031674 15286035

[28] O'Connor JC, Frame SR, Ladics GS (2002) Evaluation of a 15-day screening assay using intact male rats for identifying antiandrogens. ToxicolSci 69: 92–108. 10.1093/toxsci/69.1.92 12215663

[29] Taxvig C, Hass U, Axelstad M, Dalgaard M, Boberg J, et al (2007) Endocrine-disrupting activities in vivo of the fungicides tebuconazole and epoxiconazole. Toxicological Sciences 100: 464–473. 17785682 10.1093/toxsci/kfm227

[30] Taxvig C, Vinggaard AM, Hass U, Axelstad M, Metzdorff S, et al (2008) Endocrine-disrupting properties in vivo of widely used azole fungicides. International Journal of Andrology 31: 170–176. 18067565 10.1111/j.1365-2605.2007.00838.x

[31] Wilson VS, Lambright CR, Furr JR, Howdeshell KL, Earl GL Jr (2009) The herbicide linuron reduces testosterone production from the fetal rat testis during both in utero and in vitro exposures. ToxicolLett 186: 73–77. 10.1016/j.toxlet.2008.12.017 19167474

[32] Zhang X, Chang H, Wiseman S, He Y, Higley E, et al (2011) Bisphenol A disrupts steroidogenesis in human H295R cells. ToxicolSci 121: 320–327. 10.1093/toxsci/kfr061 21427057

[33] Ohlsson A, Ulleras E, Oskarsson A (2009) A biphasic effect of the fungicide prochloraz on aldosterone, but not cortisol, secretion in human adrenal H295R cells–underlying mechanisms. ToxicolLett 191: 174–180. 10.1016/j.toxlet.2009.08.020 19733639

[34] Sanderson JT, Boerma J, Lansbergen GW, van den BM (2002) Induction and inhibition of aromatase (CYP19) activity by various classes of pesticides in H295R human adrenocortical carcinoma cells. ToxicolApplPharmacol 182: 44–54. 10.1006/taap.2002.9420 12127262

[35] Ye L, Su ZJ, Ge RS (2011) Inhibitors of testosterone biosynthetic and metabolic activation enzymes. Molecules 16: 9983–10001. 22138857 10.3390/molecules16129983 PMC6264586

[36] Quignot N, Desmots S, Barouki R, Lemazurier E (2012) A comparison of two human cell lines and two rat gonadal cell primary cultures as in vitro screening tools for aromatase modulation. ToxicolIn Vitro 26: 107–118. 10.1016/j.tiv.2011.11.004 22120136

[37] Bardsley WG, Waight RD (1978) Factorability of the Hessian of the binding polynomial. The central issue concerning statistical ratios between binding constants, Hill plot slope and positive and negative co-operativity. JTheorBiol 72: 321–372. 10.1016/0022-5193(78)90096-6 661345

[38] Goforth HW, Fernandez F (2012) Acute neurologic effects of alcohol and drugs. NeurolClin 30: 277–ix, 277-284, ix. 10.1016/j.ncl.2011.09.015 22284063

[39] Rossi GP, Seccia TM, Maniero C, Pessina AC (2011) Drug-related hypertension and resistance to antihypertensive treatment: a call for action. JHypertens 29: 2295–2309. 22002334 10.1097/HJH.0b013e32834c465d

