# Supplementary figures and images for: Concentration Addition, Independent Action and Generalized Concentration Addition Models for Mixture Effect Prediction of Sex Hormone Synthesis In Vitro
Source: PLoS One. 2013 Aug 22;8(8):e70490. doi: 10.1371/journal.pone.0070490 (PMC3750043; doi:10.1371/journal.pone.0070490)

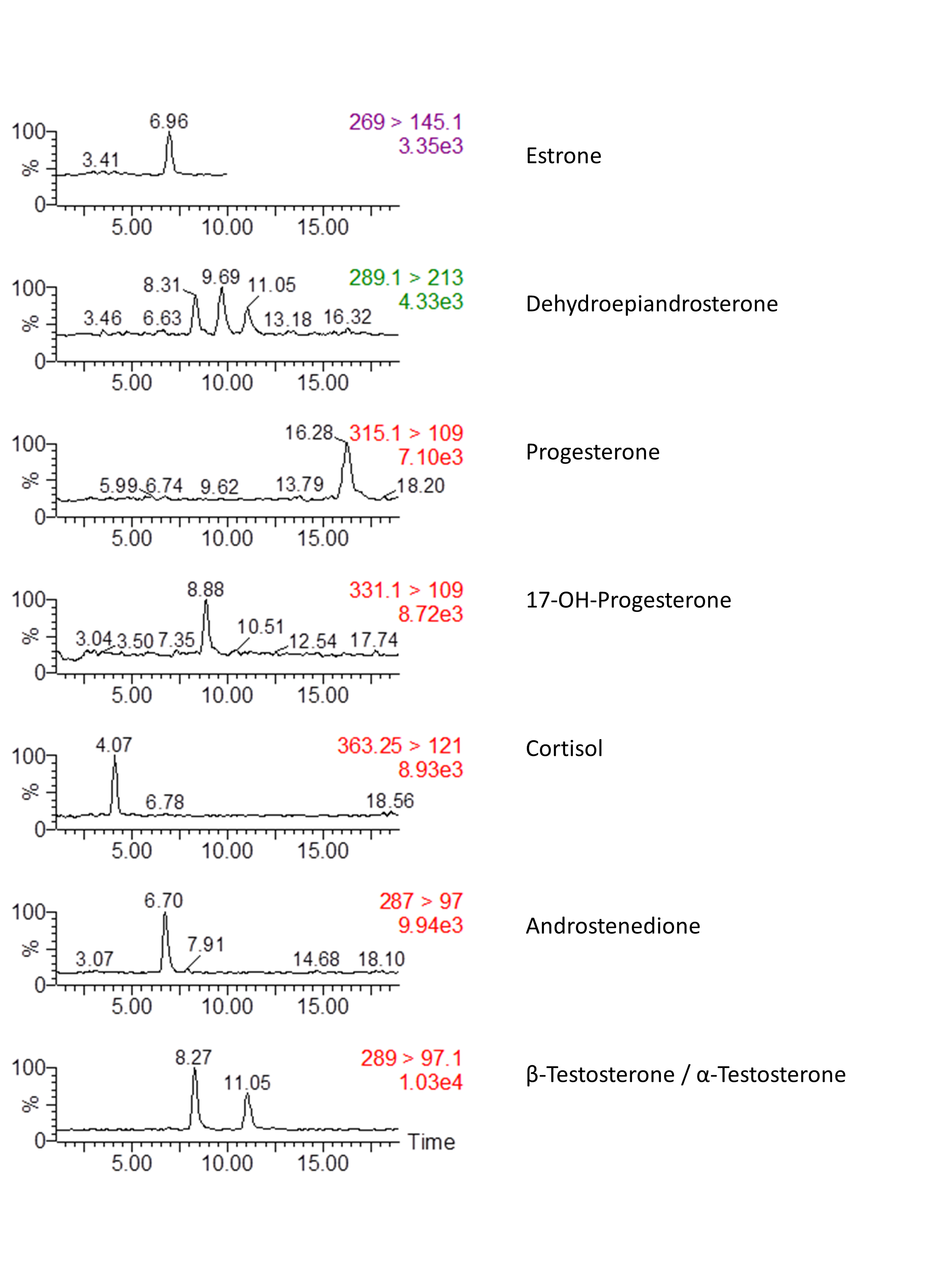

Supplement: Figure S1 — LC-MS/MS Chromatogram of standard samples used to quantify hormone levels. Estrone, progesterone, 17-OH progesterone, cortisol, androstenedione and testosterones were included at concentrations of 1.25 ng/mL. Dehydroepiandrosterone was included at a concentration of 10 ng/mL. Molecular mass of the hormones and their fragments are included in the right hand upper corner of each graph. (TIF) [file pone.0070490.s001.tif]
